# Individual endogenous pain modulation profiles within a multidimensional context of people with cervicogenic headache – A retrospective exploratory study

**DOI:** 10.1016/j.msksp.2023.102855

**Published:** 2023-10

**Authors:** Sarah Mingels, Marita Granitzer, Annina B. Schmid, Wim Dankaerts

**Affiliations:** a Musculoskeletal Research Unit, Department of Rehabilitation Sciences, Faculty of Kinesiology and Rehabilitation Sciences, Leuven University, Leuven, Belgium; b REVAL Rehabilitation Research Centre, Biomedical Research Institute, Faculty of Rehabilitation Sciences, Hasselt University, Hasselt, Belgium; c Nuffield Department of Clinical Neurosciences, University of Oxford, Oxford, United Kingdom

**Keywords:** Headache disorder, Pain modulation, Multidimensional profiling

## Abstract

**Background:**

One in four individuals with cervicogenic headache (CeH) are unresponsive to therapy. Such therapy involves predominantly biomedical interventions targeting the upper-cervical spine. A recurring theme within musculoskeletal practice is the multidimensional nature and substantial heterogeneity of the condition. Such heterogeneity might be a reason for failure of a biomedical approach. Therefore, future studies investigating efficacy of managing CeH should ideally be based on identification, and better understanding of the heterogeneity of this population based on a comprehensive evaluation of clinically relevant contributing factors.

**Objectives:**

The objective was to map profiles of individuals with CeH based on pain modulation within a multidimensional context.

**Design:**

Pain Modulation Profiles (PMPs) of 18 adults (29–51 years) with CeH were mapped retrospectively.

**Method:**

The PMPs consisted of a Pain-Profile (bilateral suboccipital, erector spinae, anterior tibialis pressure pain thresholds), a Psycho-Social-Lifestyle-Profile (Depression, Anxiety, Stress Scale, Headache Impact test, Pittsburgh Sleep Quality Index), or a combination of both. Individual results were compared to normative data. Two Pain-Profiles were defined: normal or altered. Psycho-Social-Lifestyle-Profiles were categorized based on the number of altered psycho-social-lifestyle factors (range 0–5).

**Results:**

Mapping PMPs in individuals with CeH resulted in 50% presenting with a dominant altered Pain-Profile, 16.7% with a dominant altered Psycho-Social-Lifestyle-Profile, and 5.6% with dominant alterations in both Pain-Profile and Psycho-Social-Lifestyle-Profile.

**Conclusion:**

Our results indicate heterogeneity of PMPs within the CeH population. Replication of these results is needed through dynamic assessment of the Pain-Profile before evaluating if these profiles can help patient-stratification.

## Highlights

• Pain modulation profiles are heterogeneous in the cervicogenic headache population.
• Pain and psycho-social-lifestyle profiles can be composed in cervicogenic headache.
• Pain modulation profiles are composed with minimum resources and time efficiently.
• It is required to evaluate pain modulation profiles for patient stratification.

## 1 Introduction

Cervicogenic headache (CeH) is a type of referred pain which originates from cervical structures innervated by C1–C3 spinal afferents (Bogduk and Govind, 2009). Both anatomical lesions as well as musculoskeletal dysfunctions of the upper-cervical spine could be sources of CeH (Bogduk and Govind, 2009; Núñez-Cabaleiro and Leirós-Rodríguez, 2022). Such upper-cervical musculoskeletal dysfunctions are commonly targeted by physiotherapists, manual therapists, chiropractors, and osteopaths (Biondi, 2005; Bryans et al., 2011; Luedtke et al., 2016a, Luedtke et al., 2016b; Núñez-Cabaleiro and Leirós-Rodríguez, 2022). Nevertheless, despite the well-known pathophysiology of CeH, the number of non-responders amounts to 25%, and self-reported effectiveness of manual therapy is only rated as 36% (Ossendorf et al., 2009; Liebert et al., 2013; Moore et al., 2017). Such therapy-unresponsiveness has in other musculoskeletal disorders been related to inadequate health literacy, psycho-social factors, neural sensitivity, or augmented pain processing in the central nervous system (Liebert et al., 2013; Lacey et al., 2018). Two studies confirm that pain processing could be altered in individuals with CeH (Chua et al., 2011; Mingels et al., 2021). Cephalic, but also extra-cephalic pressure pain thresholds were lower in a CeH-group compared to a matched control-group (Mingels et al., 2021). A lower level of physical activity, more stress, and a worse quality of life (QoL) were associated with these lower pressure pain thresholds (Mingels et al., 2021). Such findings might indicate heterogeneity within the general CeH-population. Meaning that CeH could present as a primarily mechanical disorder mediated by a peripheral nociceptive source (i.e. upper-cervical spine), and/or as a more complex mechanical disorder maintained by the peripheral source and processes of sensitization (Fernández-de-Las-Peñas et al., 2020). In case of the latter, clinical management needs to shift towards a multidimensional patient-centred approach including physical, psycho-social, cognitive-affective, lifestyle, and educational dimensions (Bialosky et al., 2018; Fernández-de-Las-Peñas et al., 2020). Differentiation between altered pain processing statuses might require composing a pain modulation profile (PMP) (Vaegter and Graven-Nielsen, 2016; Curatolo, 2023). PMPs are multidimensional in nature incorporating analyses of central pain mechanisms, and potential influential factors (e.g. demographic, psycho-social, lifestyle factors).

Yet, a multidimensional approach is not prioritised when managing CeH (De Pauw et al., 2021). Currently, manual therapy addressing dysfunctions of the upper-cervical spine is still mainstream in CeH-care (Luedtke et al., 2016a, Luedtke et al., 2016b; De Pauw et al., 2021). However, such management does not guarantee therapy success since a general one-size-fits-all approach might not be beneficial within a heterogeneous population (Chaibi and Russell, 2012; Garcia et al., 2016; Luedtke et al., 2016a; Fernandez et al., 2020; Mingels et al., 2021). Individual rehabilitation goals likely differ between individuals presenting with more centrally driven pain processing, and individuals without such complication (Zeppieri and George, 2017). Each individual might have personal preferences regarding rehabilitation outcomes which relate to their profile (Zeppieri et al., 2020). For example, individuals with a more mechanical-dominant CeH might benefit from focusing on physical outcome goals (e.g. range of motion, pain, strength). However, individuals presenting with CeH in combination with modifiable psycho-social-lifestyle factors (e.g. bad sleep quality, stress) may benefit more from focusing on these factors. The current biomedical approach might therefore be insufficient to personalize the intervention to specific outcomes (Zeppieri et al., 2020). In spite of arguments supporting a multidimensional approach when managing some individuals with CeH, clinical indicators studied are still based on the fact that CeH is a homogenous syndrome (Bogduk and Govind, 2009; Luedtke et al., 2016b; De Pauw et al., 2021).

In summary, variations in treatment efficacy for CeH could be driven by heterogeneity. It is suggested that different individual profiles exist (Karayannis et al., 2016; Van Dieën et al., 2019; Mingels et al., 2021). As a first step, the objective of the current study was to map the individual PMP of individuals with CeH within a multidimensional context. An exploratory analysis will be used to evaluate whether this approach should be expanded (i.e. dynamic pain modulation assessment) to a larger cohort.

## 2 Materials and methods

### 2.1 Research question

Can different PMPs be mapped among individuals with CeH?

### 2.2 Design

Retrospective profile analysis among individuals with CeH versus healthy matched controls. Data from *(blinded for review)* previously published cross-sectional study were used to compose the PMP (Mingels et al., 2021).

### 2.3 Sample size

The study is an exploratory post-hoc analysis of a published cross-sectional study (Mingels et al., 2021). We therefore did not perform a sample size or power calculation, as this would be unethical and incorrect statistical practice (Hoenig and Heisey, 2001).

### 2.4 Participants and ethics

Individuals with CeH were recruited from the headache departments of the *(blinded for review).* Inclusion criteria for individuals with CeH were: *(blinded for review)* between 18 and 55 years, body mass index between 18.5 and 24.9 kg/m^2^, diagnosed by a neurologist with CeH according to the International Classification of Headache Disorders-3 (ICHD), normal cognitive capacity (Mini Mental State Examination test score of 30) (Tashani et al., 2017; Headache Classification Committee of the International Headache Society, 2018). Exclusion criteria were: pregnancy, physiotherapy for head- or neck-related disorders in the past month before the start of the study, confounding medical pathologies (musculoskeletal, respiratory, neurological, endocrine, cardiovascular, psychiatric), comorbid headache, medication overuse (intake of non-steroidal anti-inflammatory drugs, opioids, acetylsalicylic acid, triptans, simple analgesics for >10 days/month >3 months), smoking, history of neck/head trauma, orthodontics (Appendix A). The 18 enrolled participants with CeH were given a four-week paper headache diary (Table 2) (Belgian Headache Society, 2019). Eighteen healthy asymptomatic controls were matched based on sociodemographic characteristics (age, gender, body mass index, socioeconomic status) to compose a control group (Mingels et al., 2021).

The study was based on phase 1 of a larger project which was registered as an observational study at ClinicalTrials.gov *(blinded for review)*. The *(blinded for review)* granted approval to execute the experimental protocol. Eligible participants had to read and sign the informed consent before officially being enrolled. All test procedures involving human participants were in accordance with the ethical standards of the institutional research committees and with the 1964 Helsinki Declaration and its later amendments.

### 2.5 Measurements, outcomes and instruments

**Fig. 1:**
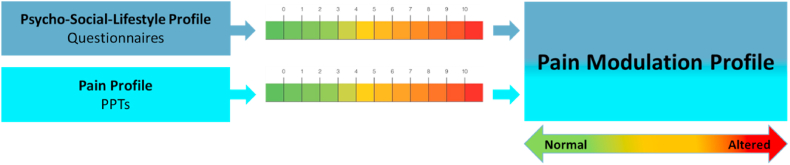
Visualization of the individual profile composition (PPTs = Pressure Pain Thresholds).

Measurements of bilateral cephalic (suboccipital muscles) and extra-cephalic (erector spinae at L1, tibialis anterior muscles) pressure pain thresholds, and questionnaires to estimate depression, anxiety, stress, (Depression Anxiety Stress Scale), QoL (Headache Impact Test), and sleep quality (Pittsburgh Sleep Quality Index) were used to compose a PMP. This profile thus comprises a Pain Profile and Psycho-Social-Lifestyle Profile (Fig. 1).

#### 2.5.1 Pain modulation profile: Pain Profile

Extra- and cephalic Pressure Pain Thresholds (PTTs) (kPa/cm^2^/sec) were bilaterally measured over the suboccipital, erector spinae (L1), and tibialis anterior muscles. PPTs were measured with an electronic pressure algometer (Somedic AB, Stockholm, Sweden) (Ylinen et al., 2007; Walton et al., 2011; Koppenhaver et al., 2015; Balaguier et al., 2016; Castien et al., 2018). The PPT is defined as the minimal amount of pressure that elicits pain. Hypersensitivity over remote, extra-cephalic sites was considered a sign of facilitated central pain processing. Intrarater reliability of PPT-measurements at the cervical muscles are good to excellent (ICC 0.82–0.99) in patients with headache (Walton et al., 2011; Martínez-Segura et al., 2012). Intrarater reliability of PPT-measurements at the tibialis anterior and erector spinae (L1) muscles are excellent in patients with neck pain (ICC 0.97) (Walton et al., 2011). PPT-measurements were executed twice by the principal researcher. ICCs ranged between moderate (ICC 0.69 suboccipital left), good (ICC 0.87 suboccipital right, ICC 0.82 tibialis anterior right), and excellent (ICC 0.94 erector spine left, ICC 0.93 erector spine right, ICC 0.92 tibialis anterior left) (Mingels et al., 2022). Averages were recorded.

Categorizing PPTs into a Pain Profile was based on cut-off points derived from the matched control-group to determine normal and altered responses. Individual PPTs lower than the 95% confidence interval lower border bound of the normative PPTs were considered as decreased (= altered); PPTs were classified as normal if they were higher than the 95% confidence interval lower border bound of the normative PPTs (Mingels et al., 2021). An individual Pain Profile was defined as ‘altered’ if all PPTs of the bilateral extra- and cephalic muscles were altered. A dominant altered Pain Profile was determined if all PPTs, and less than two psycho-social-lifestyle factors were altered (Table 1).

**Table 1:** Summary of the interpretation of a normal and dominant altered PMP.

Normal PMP	Dominant altered PMP		
	Pain	Psycho-Social-Lifestyle	Pain & Psycho-Social-Lifestyle
No altered PPTs	6 altered PPTs	>2 altered PSL factors	6 altered PPTs
No altered PSL	<2 altered PSL factors	<6 altered PPTs	>2 altered PSL factors

PMP = Pain Modulation Profile; PPTs = Pressure Pain Thresholds; PSL = Psycho-Social-Lifestyle.

**Table 2:** Sociodemographic and headache characteristics of the participants with CeH (n = 18) and the matched control group (n = 18).

	CeH group	Control group
**Age (y), mean (SD)**	40.2 (10.9)	39.2 (13.1)
[95% CI]	[34.6; 45.8]	[32.7; 45.7]
**BMI (kg/m**^**2**^**), mean (SD)**	23.5 (3.2)	23.2 (3.2)
[95% CI]	[21.9; 25.1]	[21.6; 24.8]
**Marital status, n (%)**		
Married	9 (50.0)	9 (50.0)
Living together	5 (27.8)	4 (22.2)
In a relation (not living together)	2 (11.1)	3 (16.7)
Single	2 (11.1)	2 (11.1)
**Socioeconomic status, n (%)**		
*Job*		
Student	2 (11.1)	3 (16.7)
Working	16 (88.9)	15 (83.3)
Services	14 (87.5)	13 (72.2)
Self-employed	2 (12.5)	2 (12.5)
*Level of education*		
Secondary studies	2 (11.1)	2 (11.1)
Graduate school or university	16 (88.9)	16 (88.9)
**Headache characteristics**		
Duration, mean hours/episode (SD) [95% CI]	4.1 (1.6) [3.3; 4.9]	N/A
Intensity, mean VAS/episode (SD) [95% CI]	60.9 (14) [54.4; 67.4]	N/A
Frequency, median days/month [IQR]	11 [10; 15.8]	N/A
Neck pain (yes), n (%)	18 (100)	N/A

CI = Confidence Interval; y = years; n = number participants; VAS = 100 mm Visual Analogue Scale (0 = no paint, 100 = worst pain); IQR = 25–75% Interquartile Range. Data on headache characteristics were deducted from a four-week headache-diary (Belgian Headache Society, 2019).

#### 2.5.2 Pain modulation profile: Psycho-Social-Lifestyle Profile

*The degree of depression, anxiety and/or stress* was estimated by the Dutch Depression Anxiety Stress Scale-21 (DASS-21), a self-reported one-week recall questionnaire (Lovibond and Lovibond, 1995; de Beurs et al., 2001; Parkitny et al., 2012). Each of the sub-scales contain seven items. The depression subscale assesses dysphoria, hopelessness, devaluation of life, self-deprecation, lack of interest, anhedonia and inertia. The anxiety subscale estimates autonomic arousal, skeletal muscle effects, situational anxiety, and subjective experience of anxious affect. The stress subscale evaluates difficulty in relaxing, nervous arousal, and being easily upset and impatience. Items are scored on a Likert-scale (0 = ‘Did not apply to me at all’, and 3 = ‘Applied to me very much or most of the time’). Scores of 14, 10, and 19 indicate at least moderate depression, anxiety, and stress, respectively. See Lovibond and Lovibond (1995), and Appendix B for information on the psychometric properties, and interpretation of the scores, respectively.

Impact of headache on *quality of life* was assessed with the Dutch Headache Impact Test-6 (HIT-6) (Kosinski et al., 2003; Martin et al., 2004; Kawata et al., 2005; Buse et al., 2012). The HIT-6 evaluates the impact of headache on daily activities: ability to function at work, school, home, and in social situations. Items are scored 6, 8, 10, 11, and 13 (6 = ‘Never’, 8 = ‘Rarely’, 10 = ‘Sometimes’, 11 = ‘Very often’, and 13 = ‘Always’). Scores exceeding 56 indicate headache has a significant impact on daily life. See Martin et al. (2004) and Kosinski et al. (2003), and Appendix B for information on the psychometric properties, and interpretation of the scores, respectively.

*Sleep quality* was assessed via the Dutch Pittsburgh Sleep Quality Index (PSQI), a standardized, valid and reliable self-reported one-month recall questionnaire (Buysse et al., 1989; Marinus et al., 2003; Mollayeva et al., 2016). The index differentiates poor from good sleepers by measuring seven components: subjective sleep quality, sleep latency, sleep duration, habitual sleep efficiency, sleep disturbances, use of sleeping medication, and daytime dysfunction. Scores on each of these components vary from 0 (‘No problem’) to 3 (‘Serious problem’). A maximum score exceeding 5/21 indicates poor sleep quality (Buysse et al., 1989; Smyth, 2008). See Mollayeva et al. (2016), and Appendix B for information on the psychometric properties, and interpretation of the scores, respectively.

Different classes of Psycho-Social-Lifestyle Profiles were individually composed based on the number of altered psycho-social-lifestyle factors. For each factor, results were obtained from the DASS-21, HIT-6, and PSQI. These results were compared to normative data (Mingels et al., 2021). Scores indicating at least: moderate depression, anxiety, stress (DASS-21), headache has a significant impact on daily life (HIT-6), or poor sleep quality (PSQI) were each considered as altered. As such, a Psycho-Social-Lifestyle Profile was composed of between 1 to maximal 5 psycho-social-lifestyle factors. A dominant altered Psycho-Social-Lifestyle was defined if more than two psycho-social-lifestyle factors, and less than six PPT-measurements were altered (Table 1).

#### 2.5.3 Normal Profile

This profile implies that the PPT-measurements, and scores on the DASS-21, HIT-6, and PSQI were not altered from the norms as outlined above (Table 1).

### 2.6 Procedure

Classification of the Pain Profiles and Psycho-Social-Lifestyle Profiles were combined into a PMP for each individual participant (Fig. 1). A PMP could be normal, dominant altered Pain Profile, dominant altered Psycho-Social-Lifestyle Profile, or a combined dominant altered Pain Profile/Psycho-Social-Lifestyle Profile.

### 2.7 Data-analysis

Descriptive statistics and content analysis were used to map PMPs. Primary analyses involved an analysis of proportions (%) of the different profiles in the CeH cross-sectional study (Mingels et al., 2021).

## 3 Results

### 3.1 Sociodemographics and headache characteristics

Eighteen participants with confirmed CeH and 18 healthy volunteers were enrolled in *(blinded for review)*. Table 2 provides a summary of their sociodemographic and headache characteristics.

### 3.2 Pain modulation profile

#### 3.2.1 Pain Profile

Individual and normative results for the PPT-measurements at the left- and right-sided extra-cephalic and cephalic muscles are summarized in Table 3. Altered dominant Pain Profiles were observed in nine participants (50%). Participants C1, C6, C7, C8, C11, C12, C13, C14, and C16 presented with left-sided and right-sided extra-cephalic and cephalic PPTs which were lower than the 95% lower border bound of the normative PPTs (Appendix C).

**Table 3:**
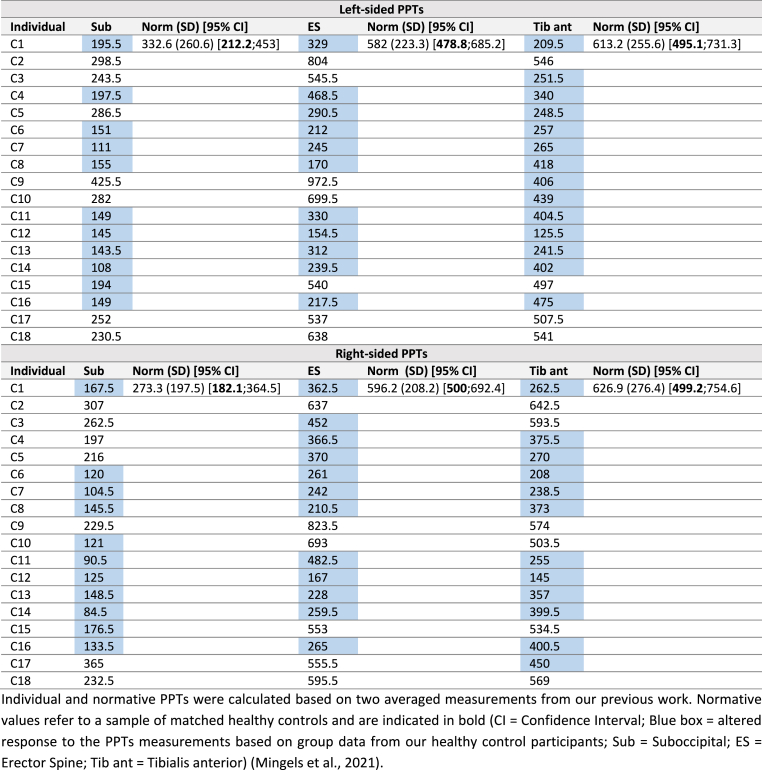
Summary of individual and normative left-sided and right-sided PPTs (kPa/cm^2^) (n = 18).

#### 3.2.2 psycho-social-lifestyle profile

Individual and normative results deducted from the questionnaires are summarized in Table 4. Five different classes of Psycho-Social-Lifestyle Profiles were observed: *Class 1* - *no altered factor*, participants C2, C3, and C16 (16.7%); *Class 2–1 altered factor*, participants C1, C12, and C18 (16.7%); *Class 3–2 altered factors*, participants C5, C7, C8, C10, C11, C13, C14, C15, and C17 (50%); *Class 4–3 altered factors*, participant C6 (5.6%); and *Class 5–4 altered factors*, participants C4, and C9 (11.1%) (Appendix C). Dominant altered Psycho-Social-Lifestyle Profiles were observed in participants C4, C6, and C9 (16.7%).

**Table 4:**
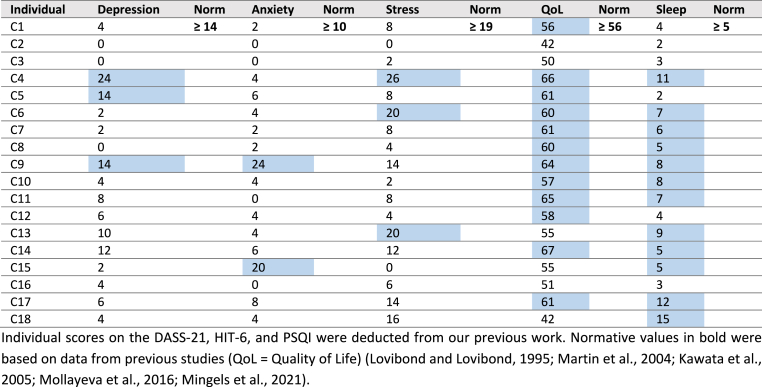
Summary of individual and normative scores on psycho-social-lifestyle questionnaires (n = 18).

#### 3.2.3 Pain modulation profile

**Fig. 2:**
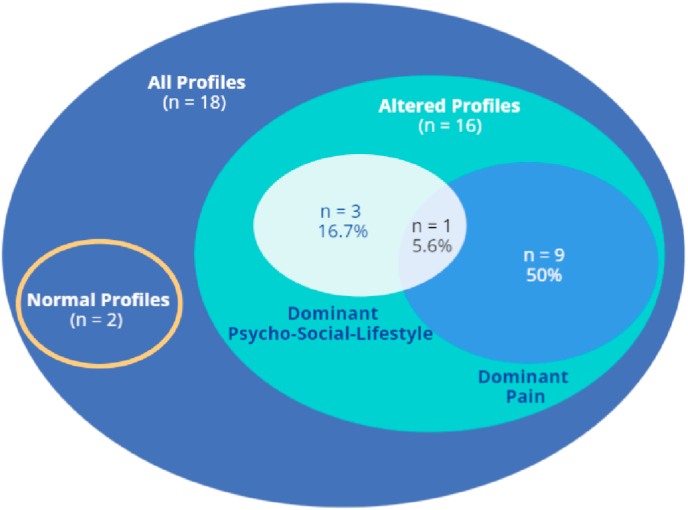
Venn diagram to visualize individual PMPs. The figure summarizes the proportion of overlap between dominant altered Pain Profiles and dominant altered Psycho-Social-Lifestyle Profiles.

Sixteen participants (88.9%) presented with altered profiles. A dominant altered Pain Profile was observed in nine participants (C1, C6, C7, C8, C11, C12, C13, C14, and C16) (50%), a dominant altered Psycho-Social-Lifestyle Profile in three participants (C4, C6, and C9) (16.7%). One participant (C6) (5.6%) presented with both a dominant altered Pain and Psycho-Social-Lifestyle Profile. Two participants (C2, and C3) (11.1%) presented with normal PMPs (Fig. 2, Appendix C).

## 4 Discussion

The objective of the current exploratory study was to map PMPs among individuals with CeH. Such mapping was feasible and resulted in 50% of the participants presenting with a dominant altered Pain Profile (i.e. six altered PPT-measurements), 16.7% with a dominant altered Psycho-Social-Lifestyle Profile (i.e. > 2 altered psycho-social-lifestyle factors), 5.6% with alterations in both the Pain and Psycho-Social-Lifestyle Profiles, and 11.1% with a Normal Profile. The results from this explorative study indicate that the population of individuals with CeH is heterogeneous.

### 4.1 Cervicogenic headache within a multidimensional context

Results from the current study suggest that individual CeH-profiles vary from musculoskeletal unidimensional, to more multidimensional profiles. Although CeH will mostly present as a musculoskeletal dysfunction of the upper-cervical spine, therapists need to be aware of the existence of a more multidimensional profile (Bogduk and Govind, 2009; Fernández-de-las-Peñas and Cuadrado, 2014; Headache Classification Committee of the International Headache Society, 2018; Fernández-de-Las-Peñas et al., 2020). Initial individual phenotyping of pain modulation should direct the management of CeH. CeH, when merely mediated by peripheral nociceptive sources of input can be managed by addressing that source through manual therapy whether or not combined with exercises (Fernández-de-las-Peñas et al., 2020). Yet, such intervention is likely inefficient if signs of facilitated central pain processing are present (an estimated 50% in our study). In such condition, exclusively aiming at the peripheral source might be insufficient (Woolf, 2011; Fernández-de-Las-Peñas et al., 2020).

Bedside quantitative sensory testing (QST) is currently the preferred proxy to assess suspected facilitated central pain processing in the absence of more direct biomarkers (Nijs et al., 2014; Arendt-Nielsen et al., 2018; Nunes et al., 2021). These complementary measurements can in the future be used to compose a PMP. Although QST can provide more detailed information on the type of sensory phenotype, it is more time-consuming, expensive, and often not readily available in clinical practice (Rolke et al., 2006; Reimer et al., 2021). Composing a PMP using the approach chosen here requires less resources and is more time-efficient.

In our study, 16.6% were classified as having a dominant altered Psycho-Social-Lifestyle Profile. The existence of this type of profile does not match the view point of international authorities, namely that CeH relates to an exclusively physical nociceptive source (Bogduk and Govind, 2009; Headache Classification Committee of the International Headache Society, 2018). Nevertheless, these findings support previous results that pain has an impact on different aspects of life, and not only on the physical component (Hagen et al., 2020). A cross-sectional survey on pain in Europe, Asia, the Americas, and Australia revealed that the impact of pain is multidimensional (e.g., impact on the QoL, physical and emotional dimensions). Accordingly, pain should be managed at the level of the individual, considering all dimensions (Hagen et al., 2020). The substantial subgroup with an altered Psycho-Social-Lifestyle Profile might therefore benefit from a targeted multidimensional approach. The complex interactions between the biological aetiology and pathogenesis, the individual, and the environment in headaches fit the biopsychosocial model. Psychological management was already reported to be beneficial in people with migraine and tension-type headache, but evidence is still lacking in people with CeH (Rosen, 2012; Rosignoli et al., 2022). A data-driven hypothetical model by Liew et al. (2023) proposes a relationship whereby psycho-physical and psychological factors result in clinical features of tension-type headache and ultimately affect disability. This data-driven model further proposes a complex relationship where poor sleep, psychological factors, and number of years with pain are relevant factors which influence disability. Research is required to determine the potential added value of targeting psycho-social-lifestyle factors in people with CeH.

A patient-centred model of care which considers all pain-relevant dimensions is needed to differentiate between the different PMPs (Schulman-Green et al., 2006; Vong et al., 2011). Better understanding of the PMP, added with the patient's individual preferred treatment outcomes could direct clinical decision-making (Elwyn et al., 2017). This approach was already successful by revealing three subgroups in patients with musculoskeletal pain (Zeppieri et al., 2020). In this context, it should be further analysed if our exploratory-based results can be used to stratify care in people with CeH.

### 4.2 Limitations

The small sample size, and retrospective nature of this study should be interpreted within the exploratory context of this study. Our findings will however inform future sample size calculations for larger studies.

Further, only static PPT-measurements were used to evaluate the Pain Profile. Such measurements provide information on one modality of somatosensation, i.e. pressure. Additional research is needed to examine if the different PMPs of individuals with CeH relate to different treatment goals, and which psycho-social, lifestyle, and sociodemographic factors are relevant within this context (Mills et al., 2019). In complex pain syndromes, patients might set treatment goals in different domains of pain in which therapists may lack confidence (Alexanders et al., 2015; Synnott et al., 2015; Gardner et al., 2017). For instance, physical therapists do not consistently check domains such as emotional distress (Oostendorp et al., 2015; Roussel et al., 2016). And, they are not very accurate and confident at identifying psychological factors (Brunner et al., 2018). Therefore, integrated psychologically informed practice is recommended to deal with more complex domains of pain (e.g. psycho-social, lifestyle) (Main and George, 2011). Such practice was developed to create a middle way between standard physical therapist practice based on biomedical principles, and more cognitive-behavioural practice originally developed to manage mental illness (Main and George, 2011).

### 4.3 Future directions - clinical translation

We propose to profile individuals with CeH based on normal or altered endogenous pain processing and psycho-social-lifestyle factors. It seems that the pathophysiology of CeH might be explained by two pain mechanisms, namely CeH caused by an exclusive peripheral input, and CeH caused by peripheral input, and maintained by a peripheral driver and facilitated central pain processes (van Griensven et al., 2020). Identifying the involved dominant pain mechanism, and additionally its associated influential factors (e.g., psycho-social-lifestyle), by composing a PMP might help patient stratification with potentially increased therapy efficacy (Main and George, 2011). Therefore, the following approach should be further explored: (1) analyse the Pain Profile and Psycho-Social-Lifestyle Profile, (2) compose the PMP, (3) question individual preferred treatment outcomes, and (4) manage the individual accordingly.

Although alterations in their PMPs were seen, 27.8% of the participants with CeH could not be assigned a dominant profile. Follow-up of these individuals might reveal transient profiles, or dynamic changes in the PMP. Further, in the current study we used depression, anxiety, stress, sleep quality, and quality of life to determine the Psycho-Social-Lifestyle Profile (Mingels et al., 2021). More research is needed into relevant prognostic sociodemographic, psycho-social-lifestyle, and cognitive-affective factors relating to disturbed endogenous pain modulation in individuals with CeH. Pain modulation should additionally be examined using dynamic protocols. This could be achieved by adding conditioned pain modulation and temporal summation paradigms that are designed to assess inhibitory and facilitatory pain pathways, respectively (Yarnitsky et al., 2014). Importantly, such measurements should be easily transferable to clinical practice (Reimer et al., 2021). Finally, the methodological approach to evaluate treatment response of a profile-based approach should be addressed.

## 5 Conclusion

This retrospective study demonstrated that individuals with CeH can be multidimensionally profiled based on pain processing and psycho-social-lifestyle factors. There was clear heterogeneity with the most frequent dominant altered profiles, i.e. the Pain Profile (50%), followed by the Psycho-Social-Lifestyle Profile (16.7%), and the combined Pain Profile and Psycho-Social-Lifestyle Profile (5.6%). Only two participants demonstrated a normal PMP (11.1%). Future work is required to understand the PMPs in more detail and evaluate their usefulness for patient stratification.

## Funding

SM is supported by the Leuven University (PDMt1/22/016). The sponsor has no role in composing the study design, collection, management, analysis, and interpretation of data, writing of the report, or the decision to submit the report for publication. ABS is supported by a Wellcome Trust Clinical Career Development Fellowship (222101/Z/20/Z) and the National Institute for Health Research (NIHR) Oxford Health Biomedical Research Centre (BRC). The views expressed are those of the authors and not necessarily those of the NHS, the NIHR or the Department of Health.

## Ethical approval

The study was based on phase 1 of a larger project which was registered as an observational study at ClinicalTrials.gov (NCT02887638). The Medisch Ethische Toetsingscommissie of Zuyderland and Zuyd Hogeschool (NL. 55720.09615), and the Comité Medische Ethiek of the Ziekenhuis Oost-Limburg (B371201423025) granted approval to execute the experimental protocol.

Eligible participants had to read and sign the informed consent before officially being enrolled. All test procedures involving human participants were in accordance with the ethical standards of the institutional research committees and with the 1964 Helsinki Declaration and its later amendments.

## Declaration of generative AI and AI-assisted technologies in the writing process

No AI tools were used during the preparation of this work.

## Declaration of competing interest

The authors have no conflict of interest to declare.
