# Supplementary material for: Individual endogenous pain modulation profiles within a multidimensional context of people with cervicogenic headache – A retrospective exploratory study
Source: Musculoskelet Sci Pract. 2023 Oct;67:None. doi: 10.1016/j.msksp.2023.102855 (PMC10560891; doi:10.1016/j.msksp.2023.102855)
Supplement: Multimedia component 1 [file mmc1.docx]

**Supplementary information**

**Appendix A - Checklist participant recruitment**

The criteria that will be used to in- and exclude participants are summarized in Table A.1.

**Table A.1** Summary of inclusion- and exclusion criteria for participants with CeH.

| Inclusion | Caucasian males, females between 18-55 years  Dutch-speaking  Fulfilment of the diagnostic criteria for secondary CeH conform the ICHD-3 or CHISG  Diagnostic conformation by a neurologist  Normal cognitive capacity (Mini Mental State Examination test score of 30).  Willing to participate |
| --- | --- |
| Exclusion | Any other headache type or headache-related disorder  Participation in another study related to headache  Pregnancy  Smoking  First headache onset > 50 years  Spinal surgery, head/neck trauma, headache-related physiotherapy ≤ 4 weeks prior the study  Post-dural punction 2 weeks prior to the study  Cognitive limitations (Mini Mental State Examination test score < 30)  ***Confounding pathologies***  - Musculoskeletal: e.g., hernia, disk prolapse, congenital spinal deviations, TMD, hypermobility (Beighton ≥ 4)  - Neurological: e.g., MS, Parkinson, epilepsy, CVA, myelopathy, myopathy, Benign Paroxysmal Position Vertigo, neurodegeneration, meningitis, encephalitis, dystonia, visual disorder  - Endocrine - System: e.g., FM, CFS, rheumatoid arthritis, infectious/inflammatory diseases, sinusitis  - Vascular: e.g., dysregulation blood pressure, postural hypotension, vertebra-basilar symptoms  - Psychiatric  - Medication-overuse: ergotamine, NSAID’s, opioids, acetylsalicylic acid, triptans, simple analgesics (> 10 days/month for > 3 months)  - Withdrawal of alcohol, caffeine or medication  - Other: orthodontics, sleep apnoea |

ICHD = International Classification Headache Disorders; CHISG = Cervicogenic Headache International Study Group Diagnostic; NSAID = Non Steroid Anti Inflammatory; MS = Multiple Sclerosis; CVA = Cerebro-Vascular Accident; FM = Fibromyalgia; CFS = Chronic Fatigue Syndrome; TMD = Temporo-mandibular disorder.

**Appendix B – Interpretation psycho-social and lifestyle factors**

Items on the ***Depression Anxiety Stress Scale-21*** are scored according a Likert-scale (0 = did not apply to me at all, and 3 = applied to me very much or most of the time). Table B.2.1 provides a summary of the interpretation of the scores on each subscale (Lovibond and Lovibond, 1995; de Beurs et al. 2001).

**Table B.2.1** Interpretation and recommended cut-off scores for the subscales of the Depression Anxiety Stress Scale-42.

|  | Depression | Anxiety | Stress |
| --- | --- | --- | --- |
| Normal | 0-9 | 0-7 | 0-14 |
| Mild | 10-13 | 8-9 | 15-18 |
| Moderate | 14-20 | 10-14 | 19-25 |
| Severe | 21-27 | 15-19 | 26-33 |
| Extremely severe | > 28 | > 20 | > 34 |

Questions on the ***Headache Impact Test-6*** are completed based on the level of agreement on a 0-4 Likert scale (never, rarely, sometimes, very often, always), and numerically converted: 6, 8, 10, 11, and 13, respectively. The impact of headache on daily life depends on the total score which varies between 36 and 78. Table B.2.2 provides a summary of the interpretation of the scores (Martin et al., 2004; Kawata et al., 2005).

**Table B.2.2** Interpretation and recommended cut-off scores for the Headache Impact Test-6.

| Score | Interpretation |
| --- | --- |
| ≤ 49 | No to little impact of headache on daily life |
| 50-55 | Headache seems to affect daily life |
| 56-59 | Headache has a significant impact on daily life |
| ≥ 60 | Headache has a very heavy impact on daily life |

The ***Pittsburgh Sleep Quality Index*** is scored 0 (no problem) to 3 (serious problem). A total score exceeding 5/21 on the Pittsburgh Sleep Quality Index indicates poor sleep quality. Table B.2.3 provides a summary of the interpretation of the scores (Mollayeva et al., 2016).

**Table B.2.3** Interpretation and recommended cut-off scores for the Pittsburgh Sleep Quality Index.

| Pittsburgh Sleep Quality Index | Interpretation | |
| --- | --- | --- |
| ≤ 5  6-7  ≥ 8 | Optimal  Borderline  Poor |  |

**Appendix C – Visualization of the results**

Figure C.1 visualizes the individual Pain Profiles. An individual altered profile was defined if all PPTs were bilaterally altered.


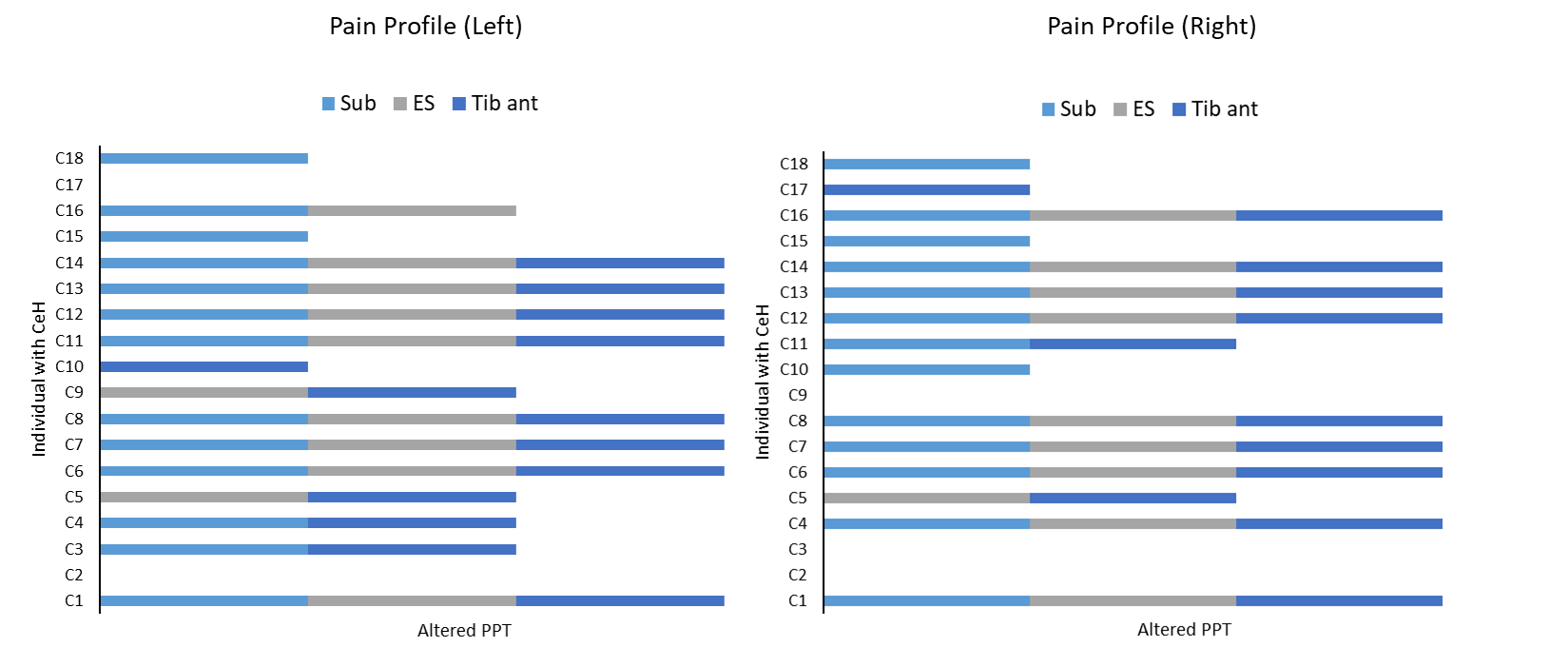


**Figure C.1.** Visualization of the individual Pain Profiles. Colored bars indicate altered PPTs as compared to the norm, the y-axis indicate the individuals with CeH (y-axis = individuals with CeH; C = individual with CeH; Sub = Suboccipital; ES = Erector Spine; Tib ant = Tibialis anterior).

Figure C.2 visualizes the individual Psycho-Social-Lifestyle Profile.


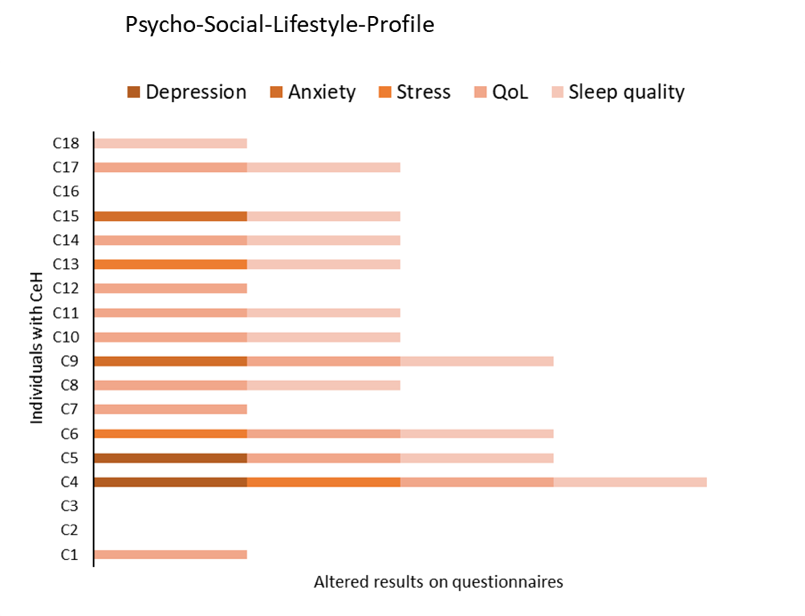


**Figure C.2.** Visualization of the individual Psycho-Social-Lifestyle Profiles. Colored bars indicate altered psycho-social-lifestyle factors as compared to the norm (y-axis = individuals with CeH; C = individual with CeH).

Figure C.3 summarizes the proportion of individual overlap between an altered Pain Profile and altered psycho-social-lifestyle factors. This figure visualizes the altered Pain Profiles supplemented with the Psycho-Social-Lifestyle Profile.


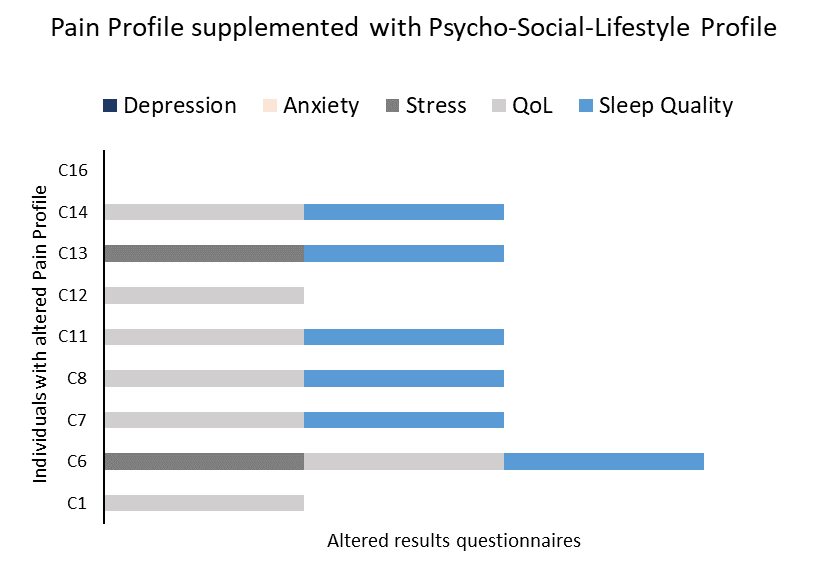


**Figure C.3.** Visualization of altered individual Pain Profiles supplemented with altered psycho-social-lifestyle factors. Colored bars indicate altered psycho-social-lifestyle factors compared to the norm (y-axis = individuals with CeH; C = individual with CeH).
